# cGAS-STING pathway targeted therapies and their applications in the treatment of high-grade glioma

**DOI:** 10.12688/f1000research.125163.1

**Published:** 2022-09-07

**Authors:** Shashwat Tripathi, Hinda Najem, Akanksha Sanjay Mahajan, Peng Zhang, Justin T Low, Alexander H Stegh, Michael A Curran, David M Ashley, Charles David James, Amy B Heimberger

**Affiliations:** 1 Department of Neurological Surgery,, Feinberg School of Medicine, Northwestern University, Chicago, IL, 60611, USA; 2 Malnati Brain Tumor Institute of the Lurie Comprehensive Cancer Center, Feinberg School of Medicine, Northwestern University, Chicago, IL, 60611, USA; 3 Department of Neurology, Feinberg School of Medicine, Northwestern University, Chicago, IL, 60611, USA; 4 Department of Neurological Surgery, Preston Robert Tisch Brain Tumor Center, Duke University Medical School, Durham, NC, 27710, USA; 5 Department of Neurological Surgery, The Brain Tumor Center, Washington University School of Medicine, St. Louis, MO, 63110, USA; 6 Department of Immunology, University of Texas MD Anderson Cancer Center, Houston, TX, 77030, USA

**Keywords:** glioblastoma, cGAS-STING, STING Agonist, immune therapies, tumor microenvironment, immune cells, myeloid cells, T cells

## Abstract

Median survival of patients with glioblastoma (GBM) treated with standard of care which consists of maximal safe resection of the contrast-enhancing portion of the tumor followed by radiation therapy with concomitant adjuvant temozolomide (TMZ) remains 15 months. The tumor microenvironment (TME) is known to contain immune suppressive myeloid cells with minimal effector T cell infiltration. Stimulator of interferon genes (STING) is an important activator of immune response and results in production of Type 1 interferon and antigen presentation by myeloid cells. This review will discuss important developments in STING agonists, potential biomarkers for STING response, and new combinatorial therapeutic approaches in gliomas.

## Introduction

Current standard of care for glioblastoma (GBM) patients consists of maximal safe resection of the contrast-enhancing portion of the tumor followed by radiation therapy with concomitant adjuvant temozolomide (TMZ). ^1^^–^^5^ Multiple factors influence GBM patient overall survival (OS), among which are age, Karnofsky Performance Score (KPS), extent of resection achieved, additional therapy received, and tumor molecular determinants such as IDH-mutation and MGMT-methylation status. ^6^^–^^13^ Despite improvements to surgical and radiation therapy, as well as the development of many novel biologic and chemical therapeutics, median survival of patients with GBM remains at approximately 15 months. ^14^^,^^15^ Among biologic therapeutics, immunotherapies (IOs), such as immune checkpoint inhibitors, have had great success in treating certain cancers, especially melanoma and lung carcinoma, ^16^^–^^20^ but have not shown success in treating GBM. ^21^^–^^23^

Analysis of the immune cell composition of the GBM tumor microenvironment (TME) has provided information that helps explain the poor response of GBM to immunotherapies. ^24^ The GBM TME is highly enriched with immune suppressive myeloid cells while lacking appreciable effector T cell infiltration. ^25^ Targeting the TME myeloid cell population to limit suppressor cell and enhance effector cell presence could be key to promoting antitumor immunity against GBM. ^26^

Stimulator of interferon genes (STING) is an important activator of immune response by promoting the production of Type 1 interferon and antigen presentation by myeloid cells, which is necessary for T cell activation and effective tumor cell killing. ^27^^–^^32^ Recent studies have revealed that the cGAS-STING pathway activity is suppressed in GBM cells ^33^ Its activation is known to contribute to anti-tumoral immune response, ^30^^,^^31^ and cGAS-STING pathway activation is therefore an intriguing objective for IOs. The recent development of potent, clinical-grade STING agonists provides an opportunity to explore STING activation as a GBM IO. ^24^^,^^31^^,^^34^^–^^38^ This review will discuss important developments in cGAS/STING pathway targeting, potential biomarkers indicative of cGAS/STING pathway activity, and new combinatorial therapeutic approaches for treating GBM that include agents for activating cGAS/STING.

### Overview of cGAS-STING pathway

**Figure 1.**
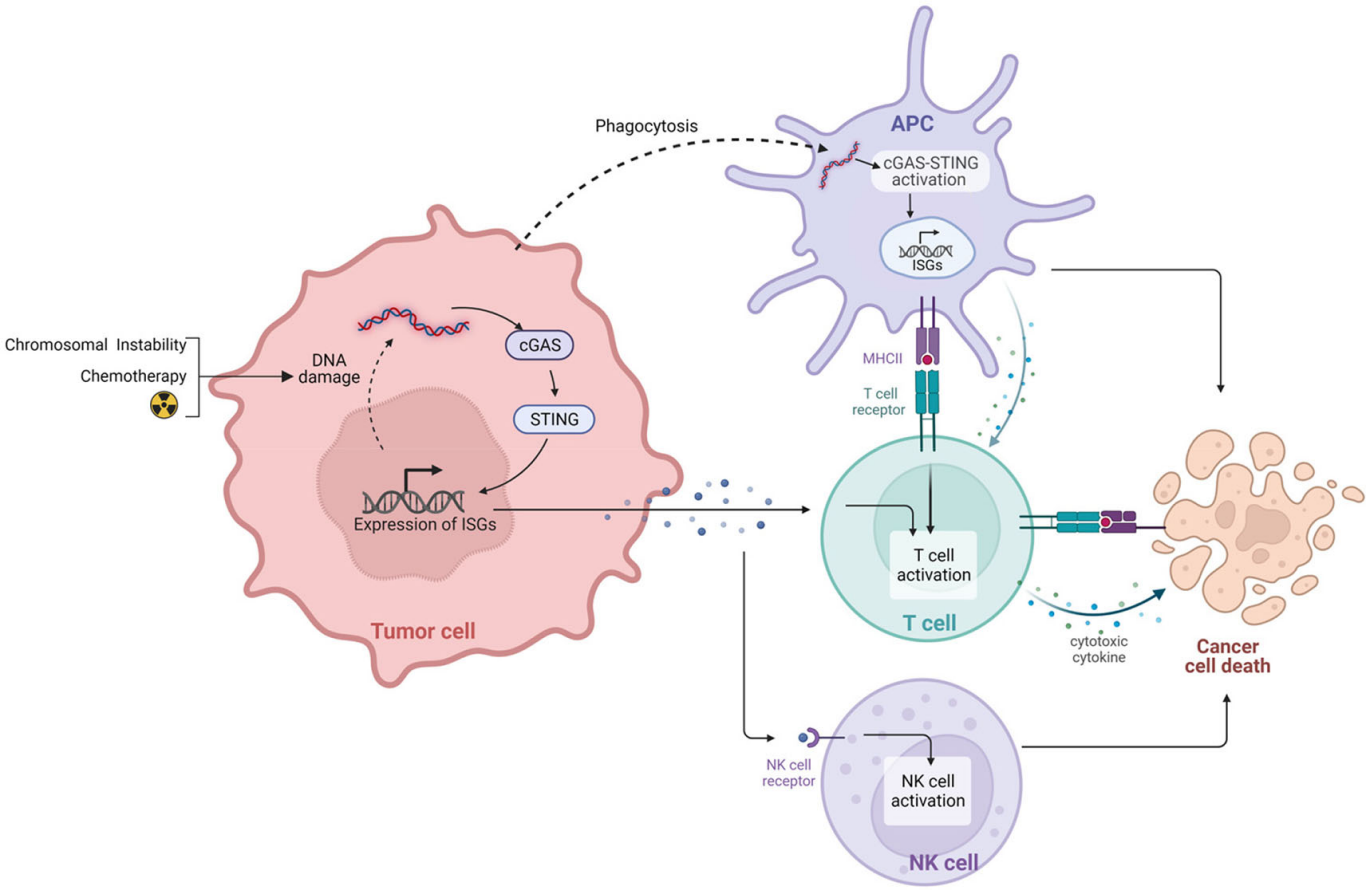
cGAS-STING activation in dying tumor cells and APCs. STING activation in antigen presenting cells (APC) occurs upon phagocytosis of tumor cells. In the phagosome, MHC loading occurs and there is subsequent activation of T cells through T-cell Receptor (TCR) signaling. Degradation of tumor cells leads to the accumulation of cytoplasmic double-stranded DNA (dsDNA). dsDNA activates cGAS triggering the conversion of ATP and GTP into cyclic GMP-AMP (cGAMP), a second messenger. cGAMP activates STING which translocates to nuclear compartment triggering transcription of interferon stimulating genes (ISGs) and production of type I IFNs and proinflammatory cytokines through phosphorylation of IRF3 and NF-kB transcription factors. Similarly, within tumor cells, cDNA accumulation occurs due to DNA damage from chromosome instability or treatment leading to cGAS-STING pathway activation. Production of type I IFNs triggers the membrane translocation of retinoic acid early transcript 1 (RAE1), a NKG2D ligand which interacts with NK Cells resulting in their activation. Created with BioRender.com.

The innate immune system relies on the binding of pathogen-associated molecular patterns or cellular damage-associated molecular patterns to Pattern Recognition Receptors (PRRs) for activation. PRRs include Toll-Like Receptors (TLRs) and DNA sensors. cGAS, a DNA sensor, recognizes intracellular cytoplasmic DNA (cytDNA) from invading microbes that have been taken up by antigen presenting cells (APCs), or that results from intrinsic genomic DNA damage ( Figure 1). Cellular damage increases circulating cDNA which can bind and activate cGAS. Activated cGAS converts ATP and GTP into cyclic GMP-AMP (cGAMP), a second messenger ^39^^–^^41^ that activates STING, also known as TMEM173. ^42^^,^^43^ Activated STING then triggers the production of type I IFNs and pro-inflammatory cytokines through IRF3 and NF-kB activation. ^27^ In multiple cancers, STING activation has been shown to repolarize macrophages from tumor-promoting M2 type to anti-tumor M1 type. ^37^^,^^44^ Recent reports have revealed that in addition to the type I IFN and proinflammatory cytokine response, STING pathway activation can lead to cell apoptosis through an IRF3-mediated mechanism. ^28^ However, this proapoptotic effect appears to be cell-type specific and is primarily triggered in T cells. ^28^ There is a need to investigate the pro-apoptotic function of STING pathway activation in the context of the GBM TME.

### Tumor induced cGAS-STING pathway activation and immune function

DNA damage, leading to increased cytDNA, can arise from either intrinsic nuclear DNA leakage due to tumor cell chromosomal instability or from DNA damage induced by therapies such as radiation or chemotherapy ^32^^,^^45^^,^^46^ ( Figure 1). cGAS pathway activation from cytDNA in tumor cells induces cellular senescence and/or intrinsic apoptotic response through second mitochondria-derived activator of caspase and capase-3. ^47^^,^^48^ Triggering of cGAS pathway activity in tumor cells activates natural killer (NK) cell anti-cancer immunity through tumor cell IFN signaling that increases cell surface presentation of NKG2D ligands such as retinoic acid early transcript 1, a structural homologue of MHC class 1, which functions as a co-stimulatory signal for NK and T cell activation. ^49^ NKG2D+ NK cells recognize and bind to NKG2D ligands expressed on tumor cells resulting in tumor cell killing. ^50^ CD8+ T cell binding also occurs to tumor cell NKG2D ligand. However, T cell activation also requires T cell receptor activation. ^51^ cGAS-STING pathway activation also occurs in TME-resident dendritic cells (DCs) that engulf circulating tumor DNA fragments or dying tumor cells ^29^ ( Figure 1). Such engulfment promotes an IFN response that triggers DC maturation and leads to stimulation of CD8+ T cell anti-tumor activity.

Knocking out cGAS or STING in a murine mammary carcinoma model has been shown to inhibit anti-tumor immune response due to inhibition of type I IFN release following radiation. ^32^ STING knock out models have also been shown to be defective in eliciting T cell response to gliomas and melanomas. ^29^^,^^31^ Conversely, STING activation inversely correlated with tumor myeloid-derived suppressor cell (MDSC) content and MDSC differentiation that can interfere with STAT3 signaling in non-myeloid cells in the TME. ^34^^,^^52^^–^^54^ In summary, cGAS-STING activation in immune cells through engulfment of dying tumor cells or tumor cell DNA leads to strong anti-tumor immunity and highlights activation of cGAS-STING as an important aspect for ensuring cancer immunotherapy efficacy.

### Targeting of cGAS-STING pathway as a cancer therapeutic

Initial preclinical studies using STING agonists involved cancer models in which agonists could be easily injected intratumorally. Results from such studies showed that local STING agonist administration reduces tumor size and/or increases survival for mice bearing melanoma, prostrate adenocarcinoma, gliomas, and head and neck squamous cell carcinomas (HNSCC). ^31^^,^^35^^,^^38^^,^^55^^,^^56^ Melanoma studies also showed that activation of STING in tumor cells and DCs increases the infiltration of NK cells in the TME due to secretion of CXCL10 and CCL5 cytokines, whereas IL-33 secretion inhibited tumor growth. ^56^ Notably, minimal CD8+ T cell activation was reported to accompany NK cell migration. ^56^

Preclinical research for activating cGAS-STING in GBM utilized a mesoporous silica nanoparticle carrying cyclic GMP to stimulate STING. ^57^ Injection of the nanoparticles resulted in complete response in 50% of flank GBM bearing mice. ^57^ In the intracranial GL261-C57BL6 GBM model, intratumoral nanoparticle injection combined with anti-TGF-β receptor 1 inhibitor administration resulted in significant extension of animal subject survival. ^57^ To increase glioma affinity and specificity Wang *et al*. developed nanoparticles loaded with STING agonist SR717 and that present RGERPPR, a glioma-specific motif. ^58^ This nanoparticle reduced tumor volume by over 50% and increased the 30-day survival rate of GL261 tumor bearing mice by more than 80%. ^58^ Analysis of the TME in animal subjects revealed STING, IRF3, and TBK1, along with increased proinflammatory cytokine levels. ^58^ Promising STING agonist results have also been observed in treating larger animal subjects. Specifically, the treatment of a canine suffering from spontaneously arising high-grade glioma with two doses of ICAS-8779 at 20 ug produced a complete tumor response. ^38^ Postmortem histopathologic analysis of the tumor showed large increases in T cells and macrophages in the TME. ^38^

With respect to cancer patient clinical trials targeting the STING pathway, most have involved the agonist 5,6-Dimethylxanthenone-4-acetic acid (DMXAA). DMXAA was originally created as a vasculature disruption agent but was incidentally discovered to directly bind to and activate STING in mouse model studies. ^59^ Subsequent studies have shown that DMXAA has a high affinity for murine STING but not for human STING, ^59^ which accounts for the mostly negative results from treating cancer patients with DMXAA ( Table 1). Limited results are available from clinical trials using other STING agonists. Results from the use of MK-1454 were initially reported at the European Society of Medical Oncology Meeting in 2018 (NCT03010176). ^60^ Treatment related adverse events occurred in 83% of monotherapy patients as well as 82% of STING agonist plus anti-PD-1 therapy treated patients. No complete or partial responses were reported in the STING monotherapy arm with 24% partial response for patients receiving dual therapy. The partial responses occurred in three HNSCC, one triple negative breast carcinoma, and two anaplastic thyroid carcinoma patients; treatment outcomes for other cancer types were not reported. In a Phase I, first-in-human multicenter open-label dose-escalation study of ADU-S100, a maximum tolerated dose was not reached, but a partial response was confirmed in one patient (2%) with Merkel cell carcinoma and stable disease was reported in 18 patients (38%) (NCT02675439). ^61^ The most common adverse events from ADU-S100 treatment were anemia, fatigue, nausea, and injection-site pain.

**Table 1.** STING agonists in clinical trials.

Agent	Indication	Phase	Co-therapy	Clinical trial	Status
ADU-S100	Advanced Solid Tumors Lymphoma	I	Anti-PD-1	NCT03172936	Terminated
		I	Anti-CTLA-4	NCT02675439	Terminated
	HNSC	II	Anti-PD-1	NCT03937141	Terminated
BMS-986301	Advanced Solid Tumors	I	Anti-PD-L1 Anti-CTLA-4	NCT03956680	Recruiting
DMXAA	Advanced Solid Tumors	I		NCT01290380	Terminated
		I		NCT01299701	Terminated
		I		NCT01278849	Terminated
		I		NCT01278758	Terminated
		I	Docetaxel	NCT01285453	Completed
		I	Carboplatin Paclitaxel Docetaxel	NCT01240642	Terminated
	Metastatic Cancer	I		NCT01278758	Terminated
	NSCLC	I	Carboplatin Paclitaxel	NCT00674102	Completed
		I/II		NCT00832494	Completed
		III		NCT00662597	Terminated
		III	Docetaxel	NCT00738387	Terminated
	Prostate Cancer	II	Docetaxel	NCT00111618	Completed
	Refractory Tumors	I		NCT00856336	Completed
	Refractory	I	Carboplatin Paclitaxel Anti-EGFR	NCT01031212	Withdrawn
	SCLC	II	Paclitaxel Carboplatin	NCT01057342	Completed
	Solid Tumors	I		NCT00003697	Completed
		I		NCT00863733	Completed
		I		NCT01299415	Terminated
E7766	Advanced Solid Tumors Lymphoma	I		NCT04144140	Recruiting
	Bladder Cancer	I		NCT04109092	Withdrawn
GSK3745417	Advanced Solid Tumors	I	Anti-PD-1	NCT03843359	Recruiting
MK-1454	HNSC	II	Anti-PD-1	NCT04220866	Active
	Advanced Solid Tumors Lymphoma	I	Anti-PD-1	NCT03010176	Active
MK2118	Advanced Solid Tumors Lymphoma	I	Anti-PD-1	NCT03249792	Recruiting
SB11285	Melanoma HNSC Advanced Solid Tumors	I	Anti-PD-L1	NCT04096638	Recruiting
SNX281	Advanced Solid Tumors Lymphoma	I	Anti-PD-1	NCT04609579	Recruiting
STAVs Loaded Leukemic Cells	Leukemias	I	Dendritic Cell Vaccine	NCT05321940	Not Yet Recruiting
TAK-50	Adenocarcinoma HCC NSCLC HNSC Mesothelioma TNBC	I	Anti-PD-1	NCT05070247	Not Yet Recruiting
TAK-676	Advanced Solid Tumors	I	Anti-PD-1	NCT04420884	Recruiting
	NSCLC TNBC HNSC	I	Anti-PD-1 Radiotherapy	NCT04879849	Recruiting

Abbreviations: HCC, Hepatocellular Carcinoma; HNSC, Head and Neck Squamous Cell Carcinoma; NSCLC, Non-Small Cell Lung Cancer; TNBC, Triple Negative Breast Cancer.

### Concerns regarding the use of STING agonists

The effects of chronic STING activation and release of proinflammatory cytokines are not fully understood. Reports of STING depletion preventing cancer metastasis and long-term STING activation maintaining cancer stemness, ^62^ promoting mesenchymal subtypes ^63^ and treatment resistance ^46^ highlight current knowledge gaps that need to be addressed to help ensure the safe and effective use of STING agonists in treating cancer patients. ^46^^,^^62^^,^^63^

In addition, research directed at drug delivery and cell population targeting needs further study. Currently, most available STING agonists must be administered intratumorally due to bioavailability limitations associated with systemic administration and poor metabolic stability. ^64^^–^^67^ In addition, systemic delivery and activation of STING can result in severe adverse side effects including autoimmunity and off-target inflammatory response. Some of the existing agonists also have relatively poor cell membrane permeability. Newer oral formulations that are being tested may overcome dose-limiting toxicities that have been reported in several clinical trials. ^66^^,^^67^ Additionally, novel delivery platforms using nanoparticles have been shown to improve stability and reduce off target effects/increase dose-response. ^58^

On important consideration when developing STING agonists are species-specific differences in STING ligand affinity and subsequent activation. ^59^^,^^68^ Most preclinical models are conducted in mice, however, structural differences between human and mouse STING have been linked to differential species-associated STING activation by DMXAA. ^59^ In addition, a single nucleotide polymorphism (SNP) in STING has been reported in humans and affects type 1 IFN production in 1–20% of the population. ^69^^–^^74^ SNPs causing STING missense variants such as R232H, which is unable to respond to c-di-AMP and 3′3′ cGAMP stimulation ^69^ indicate the need to take patient genotypes into account when considering STING agonist therapy. In total, species specific and human SNP variation need to be considered in association with STING agonist development.

Targeting of cGAS-STING has focused on the development of STING agonists while cGAS has been relatively underappreciated despite the potential of a cGAS-targeting therapeutic to more closely mimic STING signaling and produce an endogenous 2,3-cGAMP. Research examining the effects of upstream targeting of cGAS is needed. ^75^^,^^76^ Recent reports have shown that cGAMP, a product of cGAS activation, can activate DNA damage response signaling that is independent of canonical IFN pathway activity, and can induce noncanonical inflammasome pathways. ^75^^,^^76^ In murine colon cancer models cGAS deficiency has been associated with development of colon tumors, whereas STING and type I IFN receptors knockouts show no significant increase in colon tumor rates. ^77^ These results suggest cGAS as having STING-independent and cancer-relevant activities. As noted above, STING polymorphisms need to be considered when using STING agonists, whereas activation of cGAS would stimulate STING irrespective of patient-specific STING polymorphisms. Still, as is the case for the development of STING agonists, structural differences in mouse and human cGAS need to be considered to ensure proper drug development and productive outcome for the preclinical-to-clinical translation of therapeutic.

### Biomarkers of STING efficacy

For any cancer therapeutic the identification of biomarkers indicating therapeutic activity is a necessity for conducting clinical studies. Since STING mutations occur in less than 1% of all cancers, ^33^^,^^78^ cancer-associated STING variants are of a lesser concern for variations in individual tumor response to STING agonist therapy. Potential biomarkers of importance include STING promoter methylation status, patient gender, and composition of the TME including the number/percent of glioma associated myeloid cells.

Epigenetic modifications of the STING gene that influence STING expression have been shown in several cancer types as well as in STING-mediated chronic diseases. ^79^^–^^82^ Analysis of CpG promotor sites using TCGA GBM Illumina methylation array data reveal that methylation of STING cg16983159 is inversely correlated with STING expression. Analysis of STING promoter methylation status could prove important to evaluating the factors that influence cancer patient STING agonist response.

Epidemiological evidence supports a sex difference in GBM, where males have an increased prevalence and poorer outcome. ^83^ Recent studies identified differences between males and females due to genetic aberrations, cellular programs and immune response. Sex is a known variable that affects both innate and adaptive immune responses. ^84^ The Immunological Genome Project data revealed transcriptional *sexual dimorphism* in the C56BL/6J mouse where the genes of innate *immune* pathways increased before and after interferon stimulation in female macrophages. ^85^ In preclinical murine glioma models, sex specific preferential MDSC subtypes have been identified. ^86^ In the peripheral blood of female mice there are increased granulocytic (gMDSCs) and in male tumors there are increased monocytic (mMDSCs). Depletion of gMDSCs showed OS benefit in females only. ^86^ mMDSCs have been linked to radioresistance through suppression of CD8+ T cell function in preclinical colon cancer models. ^87^ As immune check point therapies have emerged, the impact of sex differences on anti-cancer immune functions has begun to be elucidated. In cancers which are responsive to immune checkpoint inhibitors (ICI), the ICI treatment is predominantly more effective in male compared to female patients. ^88^ As such, the efficacy of cGAS-STING agonists that are dependent on interferon stimulating genes (ISGs) particularly in MDCS with combinatory approaches like ICI might also be a function of sex in GBM. Pre-clinical therapeutic assessments would benefit from considering sex as a biological variable as these immunological studies may reveal differences in efficacy and sex-specific resistance mechanisms to inform clinical use.

**Figure 2.**
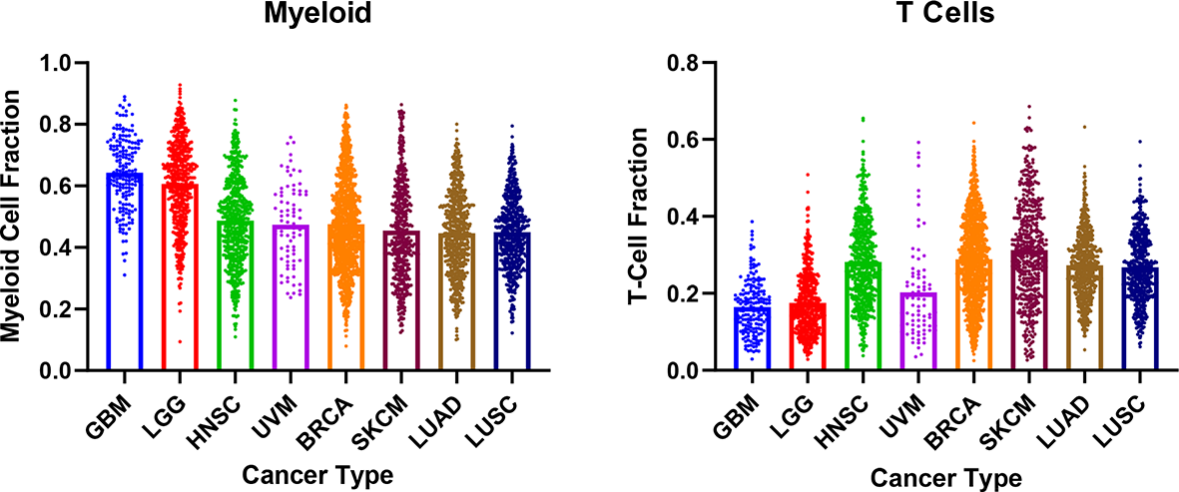
Analysis of CIBERSORT immune infiltration fractions in select cancer types. The myeloid and T cell populations in the TME were compared. Myeloid cells were defined as: Monocytes, Macrophages (M0, M1, M2), Dendritic Cells (Resting, Activated). T Cells were defined as CD8+, CD4+ (Naïve, Resting Memory, Activated Memory), Follicular Helper, and γδ. Data was obtained from Thorsson V et. al. Immunity 2018. BRCA, Breast Invasive Carcinoma; GBM, Glioblastoma; HNSC, Head and Neck Squamous Cell Carcinoma; LGG, Low Grade Glioma; LUAD, Lung Adenocarcinoma; LUSC, Lung Squamous Cell Carcinoma; SKCM, Skin Cutaneous Melanoma; UVM, Uveal Melanoma.

TME composition has been shown to influence tumor response to IO. A prototypical example involves PD-L1 expression and the presence of tumor-infiltrating lymphocytes. ^89^ For STING agonists, the presence of myeloid cell/APC infiltration in the tumor is required for anti-tumor T cell response. TME immune cell composition can be determined through immunohistochemistry or flow cytometry. ^90^ The lack of information on TME cellular composition may have been a confounder for prior Phase I clinical trials of first-generation STING agonists. There are also significant differences in myeloid and T cell infiltration within the TME when comparing across cancers, which could be important in selecting specific cancer patient populations for testing STING agonists ( Figure 2). For example, melanoma and non-small cell lung cancer are known responders to IOs and are also known for high T cell infiltration. ^16^^–^^20^^,^^91^ In contrast, GBM are preferentially enriched with myeloid cells and show infiltration by exhausted T cells that resist conversion to cytotoxic status. ^92^^–^^94^

### Combination therapies

**Figure 3.**
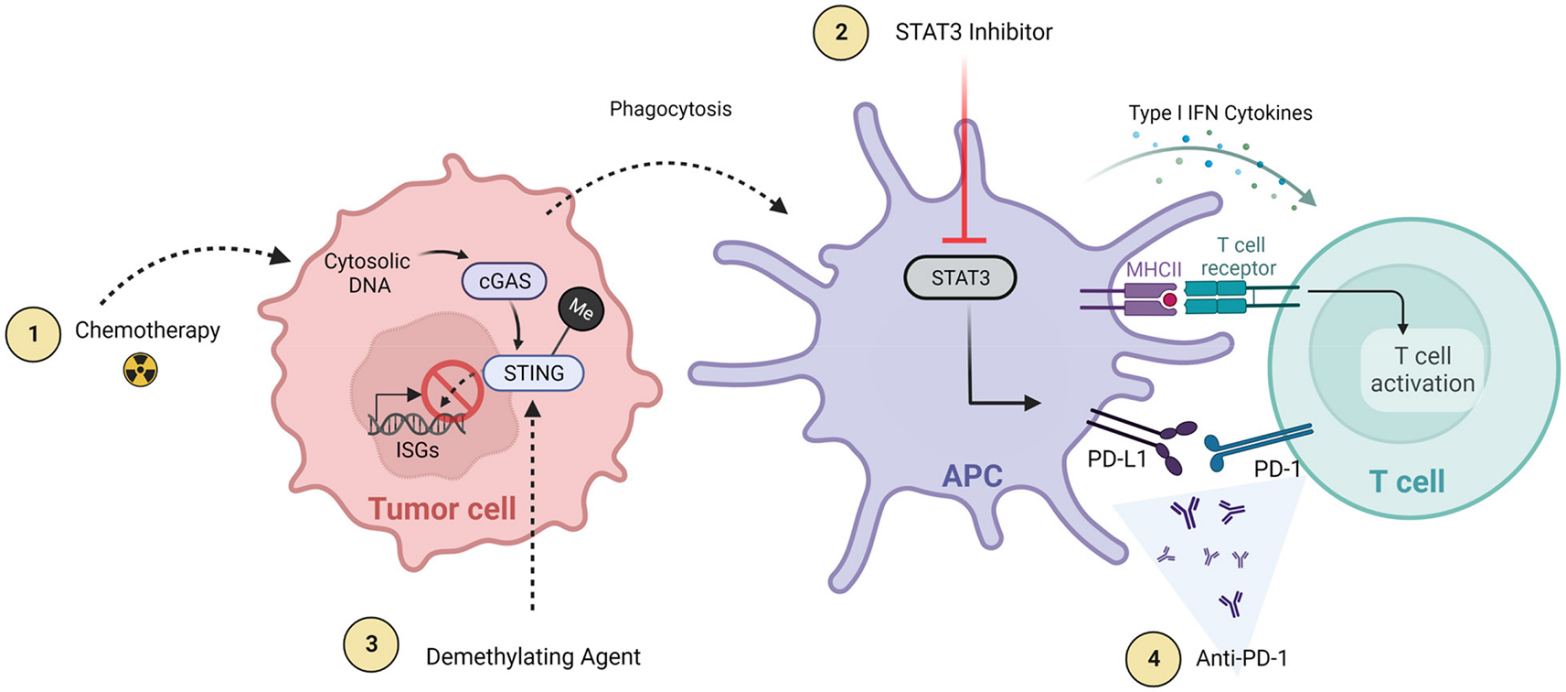
Major STING combinatorial approaches with additive or synergistic effects. 1) Radiation/chemotherapy and 2) demethylation agents that enhance downstream STING activation either through increasing cytoplasmic DNA (radiation) or decreasing STING promoter methylation (demethylating agents). Other immunotherapies (3 and 4) are synergistic therapies that focus on increasing net STING function and antitumor response through either decreasing anti-inflammatory cytokines (STAT3 inhibition) that at baseline thwart STING response or decreasing T cell deactivation and exhaustion (anti-PD-1/PD-L1). Created with BioRender.com.

Tumors evade detection and eradication utilizing a wide variety of mechanisms such as down regulation of MHC, production of immunosuppressive cytokines, and recruitment of immune suppressive cell populations. Cancers have been shown to employ these mechanisms, and others, to inhibit translocation of activated immune response mediators such as STING, through epigenetic changes (i.e., hypermethylation of the STING promoter), and by selecting for the expansion of cell with specific mutations, such as those affecting IFN receptor. ^79^^–^^82^^,^^95^^–^^98^ Due to the multiple factors promoting GBM growth, treatment for these tumors will likely require multimodal strategies targeting multiple immune response pathways. There is a growing literature supporting combination therapies that include STING pathway activation ( Figure 3).

### Demethylation approaches for enhancing STING responses

Hypermethylation of the STING promoter that reduces STING expression has been reported in gliomas, melanoma, Hepatitis C, and small cell lung cancer. ^33^^,^^79^^–^^82^ Based on such observations the combination of a STING agonist with a demethylating agent has been investigated in gliomas, melanoma, and gastric adenocarcinoma. ^33^^,^^79^^,^^81^ As a proof-of-principle demonstration, STING agonist-resistant glioma cells treated with 5-aza-2'-deoxycytidine and STING agonist responded with increased expression of IRF3 and IFIT1 that are downstream markers of STING activation. Similarly in melanoma, treatment of cells with 5-aza-2'-deoxycytidine induced phosphorylation of IRF3 and increased in CXCL10 and IFN-B following cGAMP induction. ^79^ Similar STING-relevant results have been shown for human gastric, pancreatic and colon cancer cell lines treated with zebularine (a demethylating agent) in combination with cGAMP therapy, which reduced tumor burden and extended OS for animal subjects. ^81^ Analyzing the effects of methylation inhibitors is complicated by the number of gene expressions that are influenced by the use of such inhibitors. ^99^ Nonetheless, available results highlight the promise of combining demethylating agents with STING agonists, which may be especially effective in treating hyper-methylator tumors. ^100^ Methylation analysis of the STING promoter might prove a useful biomarker for selecting patients for STING agonist therapy.

### Enhancing DNA damaging responses using STING with radiation

The effects of radiation therapy include activation of the innate and adaptive immune systems following radiation-associated DNA damage leading to robust STING-dependent and -independent type I IFN responses. ^101^ Loss of STING has been associated with decreased reactive oxygen species and DNA double strands which suggest that STING agonists may increase tumor sensitivity to ionizing radiation. ^55^ Certain preclinical study results support this possibility. For example, the combination of the STING agonist SB11285 with radiation in models of HNSCC resulted in significant decrease in tumor growth. ^55^ The combination of ADU-S100 with radiation resulted in tumor volume reduction and an increase in TME CD8+ T cells infiltration in esophageal adenocarcinoma models. ^102^ With respect to combinations involving immune checkpoint inhibitors a sharp increase in PD-L1 expression was observed in association with STING agonist treatment and thereby supporting the use of combined STING agonist and anti-PD-L1 therapy. In metastatic lung models, inhaled or intratumoral administration of nanoparticles of cGAMP (NP-cGAMP), administered with fractionated radiation therapy, showed a synergistic effect leading to robust immune response. ^103^

A potential limitation of STING agonists when used in combination with radiation is the radiation-associated recruitment of MDSCs that confer radiation resistance. ^87^ MDSCs in the TME have been implicated in poor cancer patient prognosis, metastatic disease and immunotherapy resistance. ^52^^,^^104^ Activation of type I IFN signaling might be one of the mechanisms recruiting these immunosuppressive cells within the TME. ^52^^,^^87^ To limit MDSC recruitment a CCR2 antibody could be used since CCR2 is an MDSC attractant, and CCR2 inhibition has been shown to overcome immunosuppressive radiation effects. ^87^

### APC activation while blocking STAT3 immune suppression

Tumor cells and reactive astrocytes expressing p-STAT3 release IL-6 within the TME. The released IL-6 activates STAT3 in immune cells, which neutralizes STING responses due to STAT-3 mediated production of immunosuppressive cytokines (i.e., IL-10 and TGF-β) that inhibit the production of pro-inflammatory cytokines and reduce antigen presentation. ^53^^,^^105^^–^^108^ Elevated expression of STAT3 signaling has been noted in many cancers and is correlated with poor prognosis. ^109^ As such, the combination of STING agonists with STAT3 inhibition may potentially lead to a synergistic effect and further enhance anti-inflammatory responses within the TME. ^34^^,^^52^^–^^54^^,^^107^

Several groups have examined the combination of radiotherapy, an activator of STING through increased cytDNA, and STAT3 inhibition. ^110^^–^^112^ In GBM preclinical models in which a STAT3 inhibitor was co-administered with radiotherapy, animal subjects experienced increased antigen presentation and T cell activation within the TME that extended OS. ^110^ Increased immunological synapses, defined as dendritic-T cell interactions, were identified in the combination treatment group. ^110^ Studies involving other types of cancer have also shown that combined STING agonist plus STAT3 inhibitor treatment increases the anti-tumor activity of therapy. ^53^^,^^105^ For example, in a murine breast cancer model, administration of a small molecule STAT3 inhibitor or siRNA blockade increased type I IFN signaling following treatment with STING agonist. ^53^ Alterations in the TME were observed with significant increase of CD8+ T cells combined with reduced numbers of Tregs and MDSCs. ^53^

### STING agonists in combination with immune checkpoint blockade

ICI has had little success in the treatment of GBM. Increasing tumor effector T cell infiltration and reprogramming the immunosuppressive GBM TME could increase ICI efficacy. Combinatorial use of STING and anti PD-1/PD-L1 is an attractive therapeutic strategy since STING agonists increase T cell infiltration and PD-L1 expression within the TME. ^63^^,^^102^^,^^113^^,^^114^ Additional potential synergistic effects include promoting M2 repolarization to the M1 phenotype and increasing NK cell infiltration. ^37^^,^^44^^,^^115^ This treatment approach has been evaluated in multiple preclinical studies with varying degrees of success in non-GBM cancer models. ^35^^,^^81^^,^^102^^,^^113^^,^^114^^,^^116^^,^^117^

In preclinical HNSCC models that are known to be non-immunogenic, one study showed that 80% of tumors regressed following dual therapy with STING agonist and anti-PD-L1 antibody. ^113^ Preclinical oral cancer models have shown enhanced tumor rejection and abscopal anti-tumor activity following dual therapy. ^114^ Studies combining CDN ligands with granulocyte-macrophage colony-stimulating factor (STINGVAX) and anti-PD-1 showed tumor regression in the CT26 model of murine colon carcinoma model and rejection of tumor engraftment following rechallenge, suggesting long-term tumor antigen-specific memory from earlier treatment. ^116^

Other studies have tested additional therapeutics when added onto the dual therapy backbone of STING agonist and ICB. ^35^^,^^81^^,^^102^^,^^117^^,^^118^ A three-agent therapy that included demethylating agent Zebularine increased animal subject survival and reduced tumor burden in testing multiple cancer cell lines. ^81^ STING agonists can upregulate indoleamine 2,3-dioxygenase-1 (IDO1) which promotes tumor growth, so the combination of STING agonist, IDO inhibitor, and anti-PD-1 has been investigated. ^119^ A robust anti-tumor effect has been reported from injecting a cocktail of ICB compounds (anti-PD-1, anti-CTLA-4, and anti-4-1BB) with a STING agonist, showed complete and bilateral tumor reduction in 75% of prostate cancer bearing mice. ^35^ The only side effect noted with this therapy was ulceration at the injection site that resolved within two to three weeks. A study showing increased PD-L1 expression post-treatment with combined radiation and STING agonist support future investigations to evaluate three agent therapies consisting of radiation, STING agonist, and anti-PD-1/PD-L1. ^102^

## Conclusions

STING pathway modulation is an emerging therapeutic strategy that may help overcome some of the immunotherapy limitations for treating cancers with low effector T cell levels and/or are myeloid enriched. Despite promising anti-tumor responses to STING agonists when used in preclinical studies, the risk of toxic effects from STING agonist therapy in cancer patients remains of some concern. Treatment optimization by dose de-escalations when STING agonists are used in combination therapies are under investigation. Myelosuppression and other IO-related side effects need to be weighted when creating therapeutic cocktails. Combination therapies with STING agonists may require regimen personalization to achieve benefit for each treated patient.

## Data availability

No data are associated with this article.
