# Supplementary material for: cGAS-STING pathway targeted therapies and their applications in the treatment of high-grade glioma
Source: F1000Res. 2022 Sep 7;11:1010. [Version 1] doi: 10.12688/f1000research.125163.1 (PMC9597127; doi:10.12688/f1000research.125163.1)
Supplement: Peer review history

# Peer review: Reviewer response for version 1

**DOI:** 10.5256/f1000research.137441.r151690

**Published:** 2022-10-25

**Authors:** Maria G. Castro

**Affiliations:** 1 Department of Neurosurgery, University of Michigan Medical School, Ann Arbor, MI, USA; 2 Department of Cell and Developmental Biology, University of Michigan Medical School, Ann Arbor, MI, USA

This review article by Tripathi *et al. *covers cGAS-STING pathway targeted therapies for glioma therapeutics. The review is topical, of broad interest and it is well-balanced and comprehensive. This review article will be of interest to the F1000Research readership.

 Please find enclosed below minor comments:

- The issue of blood-brain barrier penetration of the available compounds needs to be further developed. It will also be useful for the authors to discuss the timing of delivery/administration of the STING agonists in relation to the delivery of radiation therapy or chemotherapy. 
- The authors also need to add a section in relation to how the genetic makeup of the tumors could impact the efficacy of STING agonists as it is related to genomic stability. 
- Figures 1 and 3, although didactic, are very similar and convey similar messages. The authors should be encouraged to depict another aspect of the mechanism of action of STING agonists in a new figure. 
- It would be interesting to include more mechanistic details in one of the figures, on how STAT3 blockade in the tumor cells and the combination with radiation and chemotherapy could be harnessed to cooperate with STING agonists towards enhancing therapeutic efficacy.

Is the review written in accessible language?

Yes

Are all factual statements correct and adequately supported by citations?

Yes

Are the conclusions drawn appropriate in the context of the current research literature?

Yes

Is the topic of the review discussed comprehensively in the context of the current literature?

Yes

Reviewer Expertise:

Glioma therapeutics, Tumor immune microenvironment, STING pathway in gliomas, immune mechanisms in gliomas, epigenetic remodeling of the TME, scRNA-seq, epigenetics.

I confirm that I have read this submission and believe that I have an appropriate level of expertise to confirm that it is of an acceptable scientific standard.

# Peer review: Reviewer response for version 1

**DOI:** 10.5256/f1000research.137441.r149734

**Published:** 2022-10-25

**Authors:** Edjah K. Nduom

**Affiliations:** 1 Winship Cancer Institute, Emory University School of Medicine, Atlanta, GA, USA

Drs. Tripathi and all have written an extensive review detailing the rationale for the use of therapies targeting the cGAS-STING pathway in the treatment of high-grade glioma. The review is written quite well and it is not overly reliant upon review articles itself, with the authors citing many original works to support their manuscript. The article is timely as this is an emerging treatment strategy for high-grade gliomas.

 There are some relatively minor concerns with the manuscript in its current form:

- If there is any criticism, at times, it is difficult to tell when the authors are talking about the use of STING agonists generally in other cancers and metastases, and when they are specifically talking about gliomas. Much of the support for this review utilizes work in other cancers, which is a valid way to identify new treatments for gliomas, but there is not a clear transition from these articles in other cancers created a rationale for more work in STING, to this is how we are applying this approach to gliomas. I think the work would be improved if a clear progression was evident.

Is the review written in accessible language?

Yes

Are all factual statements correct and adequately supported by citations?

Yes

Are the conclusions drawn appropriate in the context of the current research literature?

Yes

Is the topic of the review discussed comprehensively in the context of the current literature?

Yes

Reviewer Expertise:

Neurosurgery, neurosurgical-oncology, immune therapy for gliomas, translational research, early phase clinical trials

I confirm that I have read this submission and believe that I have an appropriate level of expertise to confirm that it is of an acceptable scientific standard.

# Peer review: Reviewer response for version 1

**DOI:** 10.5256/f1000research.137441.r149732

**Published:** 2022-09-20

**Authors:** Marcin Kortylewski

**Affiliations:** 1 Department of Immunology, Beckman Research Institute, City of Hope Comprehensive Cancer Center, Duarte, CA, USA

This is a relatively concise but comprehensive overview of therapies targeting the cGAS/STING pathway with a specific focus on the treatment of malignant glioma. The authors are leading experts in this field with prior experience in preclinical and canine studies on STING agonists for glioma therapy. The article provides general information on this immunostimulatory pathway and then reviews the most recent advances in the development and clinical testing of STING agonists for cancer therapy.  The authors provide a rationale for targeting cGAS/STING signaling in human glioma as a monotherapy or in combination with other therapies and discuss potential biomarkers of efficacy. The review is generally well referenced and up-to-date. Thus, there are only minor comments and improvement suggestions. Overall, this is an accessible and up-to-date coverage of the rapidly progressing area of cancer immunotherapy with high value to readers from the neuro-oncology field and potentially beyond.

 Minor comments:

1. Page 3 – “Overview” section should at least briefly mention other cellular DNA sensors such as TLR9 and inflammasome complexes (e.g. AIM2, IFI16).
2. Page 3 – the use of similar abbreviations “cytDNA” and “cDNA” is a little confusing.
3. Page 6 – “Concerns” section should include recent studies linking STING signaling to unwanted effects of protumorigenic inflammation (e.g. Nature 2022 PMID: 35705809 ^1^) or brain metastasis and chemoresistance (e.g. Nature 2016 PMID: 27225120 ^2^) for a more balanced point of view.
4. Page 7 – “Biomarkers” section - the discussion of sexual differences in innate immune cells associated with glioma would benefit from including a recent study (Nat. Comm. 2021 PMID: 33608526 ^3^) which clearly indicated differences in the antigen-presenting phenotype of microglia.
5. Page 8 – the sentence “ *Analyzing the effects of methylation inhibitors is complicated by the* *number of gene expressions* ” should be rephrased.
6. Page 9 (lines 11-14) – it is important to note that IL-6 release is also the intrinsic element of STING signaling providing a negative feedback regulation, similar to the regulation of the TLR9 pathway.

Is the review written in accessible language?

Yes

Are all factual statements correct and adequately supported by citations?

Yes

Are the conclusions drawn appropriate in the context of the current research literature?

Yes

Is the topic of the review discussed comprehensively in the context of the current literature?

Yes

Reviewer Expertise:

Tumor immunology, cancer immunotherapy, oligonucleotide therapeutics

I confirm that I have read this submission and believe that I have an appropriate level of expertise to confirm that it is of an acceptable scientific standard.

## References

[1] : cGAS-STING drives the IL-6-dependent survival of chromosomally instable cancers. *Nature* .607(7918) : 10.1038/s41586-022-04847-2 366-373 10.1038/s41586-022-04847-2 35705809

[2] : Carcinoma–astrocyte gap junctions promote brain metastasis by cGAMP transfer. *Nature* .2016;533(7604) : 10.1038/nature18268 493-498 10.1038/nature18268 PMC502119527225120

[3] : Single-cell RNA sequencing reveals functional heterogeneity of glioma-associated brain macrophages. *Nature Communications* .2021;12(1) : 10.1038/s41467-021-21407-w 10.1038/s41467-021-21407-w PMC789582433608526
